# Electro-optic 3D snapshot of a laser wakefield accelerated kilo-ampere electron bunch

**DOI:** 10.1038/s41377-024-01440-2

**Published:** 2024-04-08

**Authors:** Kai Huang, Zhan Jin, Nobuhiko Nakanii, Tomonao Hosokai, Masaki Kando

**Affiliations:** 1 Kansai Institute for Photon Science (KPSI), National Institutes for Quantum Science and Technology (QST), Kyoto, Japan; 2 Laser Accelerator R&D, Innovative Light Sources Division, RIKEN SPring-8 Center, Hyogo, Japan; 3 SANKEN, Osaka University, Osaka, Japan

**Keywords:** Plasma-based accelerators, Terahertz optics

## Abstract

Laser wakefield acceleration, as an advanced accelerator concept, has attracted great attentions for its ultrahigh acceleration gradient and the capability to produce high brightness electron bunches. The three-dimensional (3D) density serves as an evaluation metric for the particle bunch quality and is intrinsically related to the applications of an accelerator. Despite its significance, this parameter has not been experimentally measured in the investigation of laser wakefield acceleration. We report on an electro-optic 3D snapshot of a laser wakefield electron bunch at a position outside the plasma. The 3D shape of the electron bunch was detected by simultaneously performing optical transition radiation imaging and electro-optic sampling. Detailed 3D structures to a few micrometer levels were reconstructed using a genetic algorithm. The electron bunch possessed a transverse size of less than 30 micrometers. The current profile shows a multi-peak structure. The main peak had a duration of < 10 fs and a peak current > 1 kA. The maximum electron 3D number density was ~ 9 × 10^21 ^m ^-3^. This research demonstrates a feasible way of 3D density monitoring on femtosecond kilo-ampere electron bunches, at any position of a beam transport line for relevant applications.

## Abstract (web summary)

The 3D density profile of a femtosecond kilo-ampere electron bunch from laser wakefield acceleration was detected by using advanced optical diagnostics and genetic algorithm.

## Graphical abstract

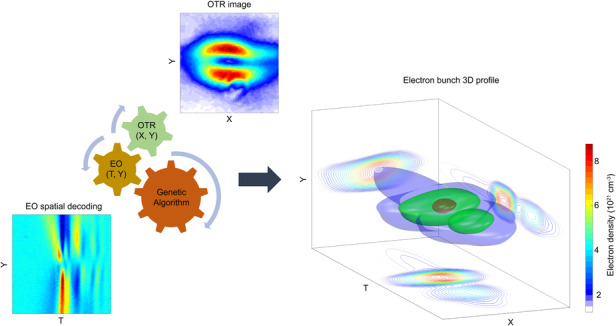

## Introduction

Accelerators have played important roles in studying high energy physics, material science, biological science, chemistry, etc. State-of-the-art accelerators based on radiofrequency (RF) cavities have sizes on the order of kilometers (km) due to the maximum sustainable acceleration gradient of a few tens of MeV m^-1^. Due to the huge cost, access to the facilities is limited. In 1979, T. Tajima and J. Dawson invented the concept of laser wakefield acceleration (LWFA)^1^. Treating a high intensity laser pulse as a bullet, the ponderomotive force drives a periodic wave in the plasma with a structure similar to an RF cavity. Electrons trapped in the wave gain energy to GeV within 1 cm, a thousand times shorter than in the case with conventional accelerators. Since the bucket size of the wake wave is merely a few tens of micrometers (μm), the electron bunches from LWFA have femtosecond temporal durations. In the last 20 years, LWFA has undergone substantial improvement^2–18^ in terms of maximum energy, energy spread, charge, repetition rate, etc.

With the high acceleration gradient and the capability of producing high brightness electron bunches, LWFA has potential applications in high energy physics, X-ray pump-probe studies and time-resolved dosimetry^19^. The three-dimensional (3D) density, *N*_3*D*_, is an important parameter affecting the luminosity in a collider, the brightness of secondary X-ray sources^20–23^ and the peak dose rates of radiation, respectively. In particular, for a tabletop X-ray free-electron laser (XFEL)^23–32^, *N*_3*D*_ is closely related to the lasing process in an undulator^24,25,33^. Until now, only the transverse^34^ or the relative longitudinal distributions^35–37^ of an electron bunch has been measured. The experimental detection of *N*_3*D*_ of an electron bunch was not conducted due to the lack of diagnostic methods.

To characterize the temporal distribution of an electron bunch, the spectral intensity analysis of transition radiation (TR)^37–40^ and the electro-optic (EO) sampling technique^41–45^ were used in conventional and laser-plasma accelerators^35–40,46–54^. The EO sampling has the merit of simultaneous detection of both the TR field strength and phase, which are indispensable for the reconstruction of the overall current profile. In an EO spatial decoding setup^41,49,50,52^, the temporal information of the bunch is imprinted onto the transverse profile of a probe laser beam. The signal field was either the Coulomb field or the TR field created by the electron bunch. The EO sampling on TR had been conducted aiming at the measurement of the relative electron longitudinal profile^35,36^ or the field strength of the terahertz (THz) pulse^55^.

The TR from an electron bunch carries the 3D information of the electrons to the far field. The image of TR in the optical range (OTR) can be used to reconstruct the transverse profile of electrons with a spatial resolution of a few micrometers^34^, which is difficult to be accomplished by detecting the fluorescence from a phosphor screen. The EO signal intensity is proportional to the TR field strength, which strongly depends on the 3D density of the electron bunch. Nevertheless, there have been few attempts to conduct a 3D polychromatic TR imaging calculation and experimentally reconstruct the absolute current profile to date.

Further, a simultaneous measurement of the transverse size and current profile after a certain distance of propagation outside the plasma is essential in LWFA because: (i) Even when the interest is solely the absolute current profile, the transverse bunch distribution at the TR source plane is indispensable in calculating the TR field strength; (ii) The bunch parameters evolve with propagation. In comparison to the *N*_3*D*_ inside or near the plasma, the *N*_3*D*_ at a specific position in the beam transport line holds greater importance for applications.

In this article, we demonstrated a snapshot of the 3D density distribution of a kilo-ampere (kA) laser wakefield accelerated electron bunch, by simultaneously performing OTR imaging and EO spatial decoding. The detailed spatial structures were reconstructed with numerical analysis using a genetic algorithm (GA). The electron transverse size was < 30 μm. The electron bunch duration was found to have a multi-peak structure. A sub-10 fs structure with a peak current of around 1 kA existed in the bunch. The peak electron 3D number density was ~ 9 × 10^21^ m^-3^ after 7 cm propagation from the exit of plasma. A preliminary discussion of how the measured 3D density can affect the experiments of LWFA-driven free electron laser was presented.

## Results

### The experimental set-up of EO 3D snapshot

**Fig. 1:**
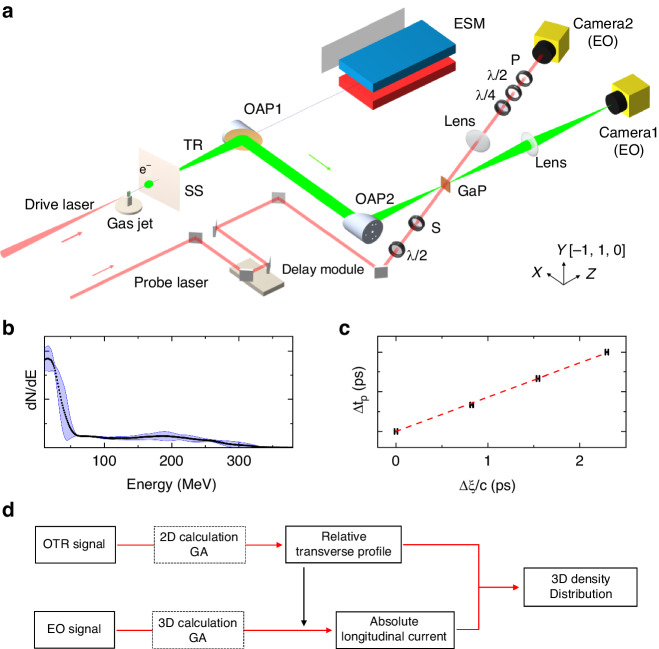
The explanation of the experiment and the concept of EO 3D snapshot. **a** Experimental setup. e^−^: electron bunch; SS: 100 µm stainless-steel foil; OAP: Off-axis-parabolic gold mirror; ESM: Electron spectrometer. The ESM was measured when removing OAP1; GaP: Gallium Phosphide crystal; S and P: A pair of nano-particle polarizers; *λ*/2 and *λ*/4: Half and quarter waveplates at 800 nm. The *λ/*2 plate was fine-tuned using a micro-actuator with an accuracy of 0.1°. The *λ/*4 was used to eliminate the residual birefringence of the EO crystal; Camera1 is for OTR imaging. Camera2 is for EO signal detection. All the items, except cameras, were placed inside the vacuum chamber. The (X, Y, Z) directions of the coordinates of TR field in the image plane is illustrated. The [-1, 1, 0] axis of the crystal and the polarization direction of the probe laser were along the “Y” direction. **b** The electron energy spectra. The black dots illustrate the averaged energy spectrum. The shaded area shows the standard deviation from statistics of five consecutive shots. **c** Confirmation of the temporal mapping relationship. ∆*t*_*p*_ is the time step in the probe delay module. ∆*ξ/c* is the peak position of the EO signal on Camera2. The error bars denote the standard deviations of the peaks of the EO signals. **d** Workflow of 3D density reconstruction

The experiment was conducted at the LAPLACIAN (Laser Acceleration Platform as a Coordinated Innovative Anchor) platform at the RIKEN SPring-8 Center, Japan. The system delivered multiple Ti: Sapphire lasers at center wavelengths around 800 nm. In this experiment, the drive laser with power up to 30 TW (1 TW = 10^12^ W) was focused onto a super-sonic gas jet to perform laser wakefield acceleration. The focal intensity of the drive laser in vacuum was 4.5 × 10^18 ^W cm^-2^. The probe laser with adjustable timings was used for EO spatial decoding. The fluence of the probe laser was tuned to be < 80 μJ cm^-2^ to avoid damage to the EO crystal^56^. The probe laser shared the same front end with the drive laser but had a separate pulse compressor. The electron beams were generated by using a 4 mm slit nozzle with a mixture gas of (hydrogen: nitrogen) = (99:1). At a backing pressure of 0.55 MPa, relatively stable electron beams were produced. The experimental set-up is plotted in Fig. 1a. The electron energy spectra were plotted in Fig. 1b. The electrons had relatively stable energy spectra with continuous profiles, due to the ionization injection^57–61^. The OTR and EO calculations in this article utilized the electron energy spectra measured in the experiment. The very slight variations of the electron spectra had negligible impact on the 3D snapshot (see “Materials and Methods” for details).

To generate TR, a stainless-steel (SS) foil with a thickness of 100 *μ*m was inserted at a position 7 cm after the gas jet. The TR was then collected to the EO crystal using an imaging system composed of two identical OAPs (focal length: 19 cm; diameter: 50.8 mm). The magnification of the 2-OAP system was *M*_*1*_ = 1. The distance between the OAPs was 40 cm. The TR transmitted from the EO crystal was imaged to Camera1 with a magnification *M*_*2*_ = 1.96. The EO crystal used in the experiment was a (110)-cut GaP crystal with a thickness of 30 μm. The probe laser polarization was aligned with the [-1,1,0] axis of the crystal. The relative angle between the probe laser and the TR line was measured to be ${\theta }_{p}^{{\prime} }$ ~ 23° from the experimental set-up. The EO signals were measured at several probe timings to confirm the temporal mapping relationship, as shown in Fig. 1c. A linear fitting shows a relation of $c\varDelta {t}_{p}=0.432\varDelta \xi$. By fitting with a widely used model: $\varDelta t=\tan {\theta }_{p}\varDelta \xi /c$, we found *θ*_*p*_ = 23.3°. The calculated *θ*_*p*_ is quite close to the measured ${\theta }_{p}{\prime}$, indicating that the TR pulse was incident normally on the surface of the EO crystal. This measurement indicated a nominal temporal resolution of 1.26 fs pixel^-1^.

### The workflow of the 3D reconstruction of the electron bunch

With the signals of OTR imaging and EO sampling, the workflow of the 3D density reconstruction is presented in Fig. 1d. 2D (*x*, *y*) calculations assisted by GA were used to retrieve the relative transverse profile from the OTR image. Combined with the obtained transverse profile, 3D (*x*, *y*, *ω* or *t*) calculations with GA were used to reproduce the EO signal, and the absolute longitudinal current profile was obtained. Finally, the 3D density was reconstructed. The transverse sizes and temporal durations of electron bunches in this article are defined in “*rms*”.

### The reconstruction of the relative transverse profile

**Fig. 2:**
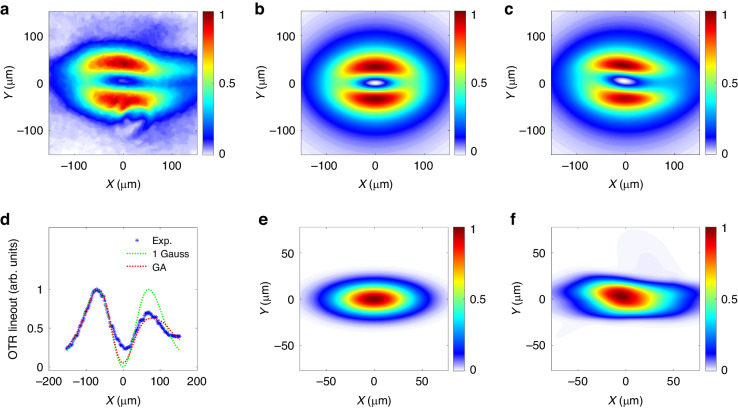
**Reconstruction of the transverse electron beam profile**. **a** Experimental OTR image. **b**, **c** are the calculated COTR images by the “1 Gauss” and “GA” models. **e**, **f** are the reconstructed transverse bunch profiles from “1 Gauss” and “GA”, respectively. **d** illustrates the horizontal lineouts at “*Y* = *0*” in the OTR images. Blue asterisks denote the experimental lineout. The green and red dots denote the calculated lineouts from “1 Gauss” and “GA”, respectively. The signal intensities are normalized with a maximum of 1

An experimental OTR image is shown in Fig. 2a. The “donut” shape is a coherent feature. For ionization injection, the electrons often have modulated time structures due to the interaction with the drive laser or the phase-dependent tunneling ionization^58–61^. Numerical simulations showed that the periodic modulations were mostly not concentrated at a specific spatial point (see references^57–60^ and the “Supplementary information”). With existing practice^34,62^, we used COTR calculations to reproduce the primary features of the experimental OTR.

We demonstrated that the calculation can be conducted using an analytical model^63,64^ with considerable accuracy^65^. The TR field at the imaging plane from a single electron can be calculated as

1 $${{\boldsymbol{E}}}_{0}^{{TR}}\left(x,y,\lambda ,\gamma \right)=-\frac{e}{{\epsilon }_{0}\lambda {vM}}\frac{\left(x,y\right)}{r}f\left({\theta }_{m},\gamma ,\zeta \right)$$

where (*x,y*) denote the image plane coordinates. $r=\sqrt{{x}^{2}+{y}^{2}}$. *θ*_*m*_ denotes the acceptance angle. $\zeta =2\pi r/\lambda M$ denotes a normalized transverse distance. *M* denotes the magnification of the imaging system. When $1/\gamma \ll {\theta }_{m}$, the diffraction factor has a simplified form: $f\left({\theta }_{m},\gamma ,\zeta \right)\approx {\gamma }^{-1}{K}_{1}\left({\gamma }^{-1}\zeta \right)-{\zeta }^{-1}{J}_{0}\left(\zeta {\theta }_{m}\right)$. Though a bandpass filter was absent, we carefully demonstrated that an average wavelength of $\bar{\lambda }$ = 550 nm can be used to simplify the calculation with good accuracy (see “Supplementary information” for details). From the experimental electron energy spectra, we obtained *γ*_*j*_ with weight *χ*_*j*_. The field point-spread function (FPSF)^66^ can be calculated as ${FPS}{F}_{x,y}=\sum _{j}{\chi }_{j}{{\boldsymbol{E}}}_{0}^{{TR}}(x,y,\bar{\lambda },{\gamma }_{j})$. In OTR calculation, we used the *θ*_*m*_ in the second stage of the imaging since it was smaller than the acceptance angle of OAP1 in the first stage. With an arbitrary transverse electron profile ${g}_{\perp }$, the relative COTR intensity is calculated by

2 $${I}_{{COTR}}={\left|{g}_{\perp }* {FPS}{F}_{x}\right|}^{2}+{\left|{g}_{\perp }* {FPS}{F}_{y}\right|}^{2}$$

where $"*"$ denotes convolution. In a previous study^66^, a relation ${\left(x,y\right)}_{\max }=A+B\cdot {\sigma }_{x,y}$ was reported using a single Gaussian (“1 Gauss”) distribution: ${g}_{\perp }(x,y)\propto \exp (-{x}^{2}/2{\sigma }_{x}^{2}-{y}^{2}/2{\sigma }_{y}^{2})$. *A* and *B* are constants. ${\left(x,y\right)}_{\max }$ are the peak positions of the central lineouts in the COTR images. The beam sizes of (*σ*_*x*_, *σ*_*y*_) = (28, 11) μm were good solutions. The lineout along the “X” direction is plotted in Fig. 2d as the green dots. The calculation of the COTR image and electron bunch profile are shown in Fig. 2b and e, respectively. Although this method can be used to roughly estimate the transverse sizes, the detailed features of the experimental OTR are not reproduced.

A simple deconvolution of the COTR is not possible because the intensity distribution does not have the form ${PSF}* {g}_{\perp }$ as in the incoherent case^63^, where ${PSF}\sim ({|{FPS}{F}_{x}|}^{2}+{|{FPS}{F}_{y}|}^{2})$ is the intensity point spread function (${PSF}\ne {FPSF}$). To solve the problem, GA was implemented. The COTR profile ${I}_{{COTR}}^{{GA}}$ and transverse electron beam profile ${g}_{\perp }^{{GA}}$ from GA are shown in Fig. 2c, f. Figure 2c and the red dotted curve in Fig. 2d demonstrate a notable enhancement in the similarity of the COTR between the calculated and experimental results. Therefore, Fig. 2f is used as the transverse profile for the calculations of the absolute TR field strength and EO signal shapes. The details of the structure designs of GA for OTR calculation can be found in the “Materials and Method”.

### Reconstruction of the absolute current profile

**Fig. 3:**
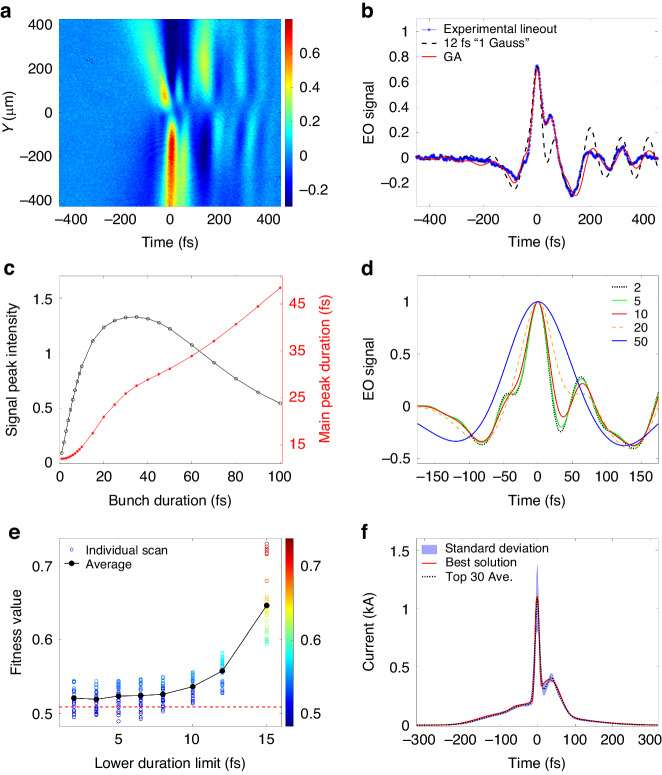
**The experimental EO signal and analysis**. **a** The experimental 2D (*t*, *Y*) EO signal from a 30 µm GaP crystal. The experimental EO signal lineouts at *Y* = -200 µm are illustrated as blue asterisks in **b**. The red and black dashed curves denote the calculated results from “GA” and a single Gaussian bunch with a duration of 12 fs. **c** Variation trend of the main peaks of the EO signals for different electron bunch durations. The black circles show the absolute peak values. The red asterisks show the *rms* durations of the main peak by conducting Gaussian fittings. **d** Normalized calculated EO signal shapes for different bunch durations. (black, green, red, yellow, blue) correspond to (2, 5, 10, 20, 50) fs, respectively. **e** shows the fitness values of various lower limit *σ*_*l*_ for GA. The colored circles are the individual scanning values. The black dots illustrate the mean values at each *σ*_*l*_. The red dashed line denotes the threshold of picking out the “Top 30” solutions. **f** shows the reconstructed current profiles from GA. The red curve illustrates the current profile from the best solution. The black dotted curve shows the averaged current profile from the top 30 GA solutions. The shaded area illustrates the standard deviation from the statistics of the top 30 solutions

The experimental EO signal is illustrated in Fig. 3a. The signal with background subtraction was ${I}_{{sig}}=\left[\cos \left(4{\theta }_{2}\right)-\cos \left(\varGamma +4{\theta }_{2}\right)\right]/\left[1-\cos \left(4{\theta }_{2}\right)+2{\delta }_{{ext}}\right]$^54,65^, where *Γ* is the phase retardation and *δ*_*ext*_ indicates the extinction rate of the polarizer pair^53,54^. The *λ*/2 plate was rotated by *θ*_2_ = 3° to perform a near-cross-polarization detection^50^. By adjusting the probe timing to achieve the highest signal intensity, EO spatial decoding occurred at “*X* ~ 0” in the coordinates of the TR field. The signal had opposite signs relative to “*Y* = 0”, in correspondence with the radial polarization of the TR field. The signal is broadened further from the center of the TR field. We proceeded by checking *I*_*sig*_ at a specific spatial point (*X*, *Y*) = (0, -200) μm, as indicated by the blue asterisks in Fig. 3b. The oscillations in Fig. 3a and b are results from the absorption and phase-mismatch inside the EO crystal, when the TR field has femtosecond durations and contains considerable high frequency components^50,51,54,65^.

To confirm the detection limit, the EO signals from a 30 μm GaP crystal were calculated with electron bunch durations ranging from 1 to 100 fs (with a fixed peak current of 1 kA). Using the bunch transverse profile shown in Fig. 2f, we calculated the 3D frequency domain (from THz to near-infrared) TR electric field ${E}^{{TR}}\left(x,y,\omega \right)$ and the EO spatial decoding. With an arbitrary normalized temporal profile ${g}_{z}\left(t\right)$, the longitudinal form factor is ${F}_{z}\left(\omega \right)=\int {g}_{z}\left(t\right){e}^{i\omega t}{dt}$. The ${E}_{y}^{{TR}}$ contributing to phase retardation is calculated by

3 $${E}_{y}^{{TR}}\left(x,y,\omega \right)={g}_{\perp }\left(x,y\right)* \mathop{\sum }\limits_{j}{\chi }_{j}{E}_{0,y}^{{TR}}\left(x,y,\omega ,{\gamma }_{j}\right){F}_{z}\left(\omega \right)$$

The temporal energy chirp is omitted because it has a negligible effect on the EO signal^65^. Although ${E}_{y}^{{TR}}\left(x,y,\omega \right)$ has a transverse distribution, we demonstrate that when the interested time region is small, the calculation can be performed using the TR field information at a specific spatial point with extremely small error^65^. Considering the relative angle between the probe and the signal field in EO spatial decoding, the phase retardation encoded to a probe with an infinitesimal duration is derived as^65^:

4 $$\begin{array}{c}{\varGamma }_{0}\left(\tau \right)=\frac{{n}_{0}^{3}}{{\lambda }_{0}}\int d\omega \frac{2}{1+N\left(\omega \right)}{E}_{y}^{{TR}}\left(\omega \right){r}_{41}\left(\omega \right)\\ \times {\int }_{0}^{d}\exp \left[i\omega \left(\frac{N\left(\omega \right)}{c}-\frac{1}{{v}_{\parallel }}\right){dz}\right]\end{array}$$

where ${n}_{0}$ and ${\lambda }_{0}$ denote the refractive index and wavelength of the probe, respectively. $N\left(\omega \right)$ and ${r}_{41}\left(\omega \right)$ denote the complex refractive index and EO coefficient of the TR pulse^50^. *d* is the thickness of the crystal. ${v}_{\parallel }$ is the probe group velocity component along the propagation direction of the TR field inside the crystal. The phase retardation was then calculated as a correlation: $\varGamma ={\varGamma }_{0}\star \bar{{I}_{p}}$, where $\bar{{I}_{p}}$ is the averaged probe temporal profiles inside the crystal.

The absolute peak values and fitted durations of the main peaks of the EO signals are shown in Fig. 3c. The smaller signal intensities at the short and long duration ends can be attributed to the lack of EO response at short wavelengths and diffraction loss of the TR field at long wavelengths, respectively. The normalized EO signal shapes (with the peak normalized to 1) are plotted in Fig. 3d with electron bunch duration ${\sigma }_{t}$ of {2, 5, 10, 20, 50} fs. For very short electron bunches, a characteristic side peak at similar timings exists, due to the optical properties of the GaP crystal. The signal shapes have small differences for electron bunch durations even down to a few femtoseconds (see “Materials and Methods” for details). However, the signal intensities decrease significantly, as illustrated by the black circles in Fig. 3c. The peak signal intensity of 5 fs electron bunch ${I}_{{sig}}^{5{fs}}$ is compatible with ${I}_{{sig}}^{100{fs}}$. Yet, the peak of ${I}_{{sig}}^{1{fs}}$ is one fifth of ${I}_{{sig}}^{5{fs}}$. Due to this limitation, the EO sampling in our experiment was not sensitive to the sub-fs spikes. The lateral analysis focuses on the envelope of the absolute current profile formed by a major part of the electrons.

The experimental signal in Fig. 3b had a main peak duration of 16 fs, followed by a side peak at ~ 50 fs. By verifying the relationship illustrated by the red curve in Fig. 3c, the main peak of the experimental EO signal corresponded to an electron bunch duration ~ 12 fs. The calculated EO signal with a 12 fs electron bunch is plotted as the black dashed curve in Fig. 3b. Although the main peak fitted well, the characteristic side peak caused by the absorption and phase mismatch in the GaP crystal exists around 65 fs (> 50 fs). This discrepancy suggests that the electron temporal profile should be more complicated than just a single Gaussian shape.

Direct deconvolution is difficult because the noise is high at frequencies where ${r}_{41}$ crosses zero. To overcome this challenge, genetic algorithm (GA) was employed to reconstruct the detailed temporal structure. Given the slight variations in electro-optic (EO) shapes even when electrons exhibit sub-10 fs durations (as detailed in “Materials and Methods”), we conducted a scan on the lower limit of the electron bunch duration ${\sigma }_{l}$, as shown in Fig. 3e. 30 rounds of GA calculations were performed with ${\sigma }_{l}$ at each of {2, 3.5, 5, 6.5, 8, 10, 12, 15} fs. The average fitness values decreased (better) quickly when ${\sigma }_{l}$ shifted from above-10 fs to sub-10 fs region. However, the differences in the mean values for ${\sigma }_{l}\in$ {2, 3.5, 5, 6.5} fs were not pronounced.

The *I*_*sig*_ from the GA solution with minimum fitness value is plotted as the red curve in Fig. 3f. The experimental signal shapes around the main and side peaks are well reproduced. The current profile of the best solution exhibits a peak current value of 1.1 kA. It has a multi-peak structure. The main peak has a duration of ~ 6.5 fs. To increase the confidence of the results, statistics were conducted on the top 30 solutions (the solutions with fitness values below the red dashed line in Fig. 3e) from all the scanning results. The standard deviations of the current profiles are illustrated by the blue shaded area in Fig. 3f. The averaged current profile is plotted as the black dotted curve. It also has a multi-peak structure with a main-peak duration of ~7 fs and a peak value of 1.09 kA. Though slight differences exist between the best and averaged curves, both profiles share similar pedestals and main peaks, and have similar locations of side current peak around 35 fs after the main current peak. The details of the structure designs of GA for EO calculation can be found in the “Materials and method”.

### The reconstruction of the electron 3D number density and the impact to the FEL experiment

**Fig. 4:**
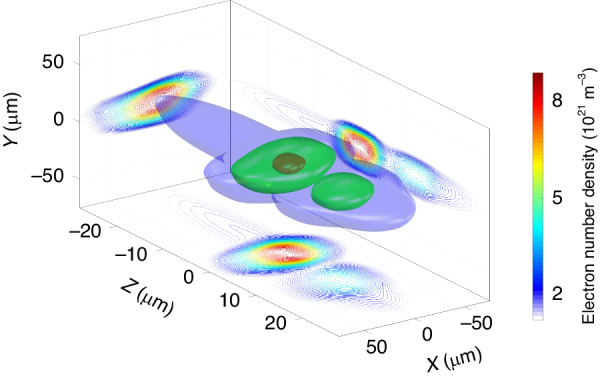
**3D number density profile of the electron bunch**. *Z* = *ct* represents the longitudinal dimension, where “-” denotes the forward direction. Slice plots at the side wall were conducted at plane of (*X* = 0), (*Y* = 0) and (*Z* = 0), respectively. For the volume plot, blue, green, and red colors denote contour values of {*e*^−2^, *e*^−1^, 0.9} of the maximum

With the transverse profile in Fig. 2f and the absolute current profile in Fig. 3f, the 3D number density distribution is reconstructed and plotted in Fig. 4. The electron bunch has a peak number density ${N}_{3D}^{{peak}}$∼ 9 × 10^21^ m^−3^.

We take the LWFA-driven FEL as an example to elaborate on how the measured 3D density value can affect the application of LWFA. In FEL, the Pierce parameter^24^ is $\rho ={\left(\frac{1}{16}\frac{{I}_{e}}{{I}_{A}}\frac{{K}_{0}^{2}{\left[{JJ}\right]}^{2}}{{\gamma }_{0}^{3}{\sigma }_{x}^{2}{k}_{u}^{2}}\right)}^{1/3}$ and the 1D gain length is ${L}_{G0}={\lambda }_{u}/\left(4\pi \sqrt{3}\rho \right)$. $\left[{JJ}\right]=\left[{J}_{0}\left(\xi \right)-{J}_{1}\left(\xi \right)\right]$ and $\xi ={K}_{0}^{2}/\left(4+2{K}_{0}^{2}\right)$. ${K}_{0}=e{B}_{0}/\left({mc}{k}_{u}\right)$ is the dimensionless undulator strength parameter. ${k}_{u}=2\pi /{\lambda }_{u}$ is the undulator wavenumber. ${\lambda }_{u}$ is the undulator period. *I*_*A*_ ~ 17 kA is the Alfven current. It can be readily found that the factor ${I}_{e}/{\sigma }_{x}^{2}$ corresponds to the 3D charge density of the electron bunch. Without loss of generality, by assuming a 3D Gaussian shape, the peak current is related with the total charge and electron number as ${I}_{e}=Q/\left(\sqrt{2\pi }{\sigma }_{t}\right)=N{e}_{0}c/\left(\sqrt{2\pi }{\sigma }_{z}\right)$, where ${\sigma }_{t}={\sigma }_{z}/c$, *N* is the electron number and *e*_0_ = 1.6 × 10^-19^ C. The Pierce parameter can be rewritten as:

5 $$\rho ={\left(\frac{1}{16}\frac{{N}_{3D}^{{peak}}2\pi c{e}_{0}}{{I}_{A}}\frac{{K}_{0}^{2}{\left[{JJ}\right]}^{2}}{{\gamma }_{0}^{3}{k}_{u}^{2}}\right)}^{1/3}$$

We use $\left({K}_{0},{\lambda }_{u}\right)$ = (1.4, 25 mm) in the following calculation. In the LWFA-driven FEL experiment, the electron bunches need to be delivered to the undulators through a beam transport line. The electron bunches evolve in phase space during a few meters of propagation. At the entrance of the undulator, the electrons have a transverse size of a few hundred micrometers or millimeters^29,30,32^. For a transverse size of 1 mm, even without temporal elongation, the maximum density ${N}_{3D}^{{peak}}$ will drop from 9 × 10^21 ^m^-3^ to 2.8 × 10^18 ^m^-3^. Assuming the electrons have zero energy spread, we calculated the FEL parameters for electron energies of {100, 500, 1000} MeV. The necessary undulator length *L*_*u*_ was estimated to be 20 times of the gain length for the operation of FEL in the saturation regime^67^. For comparison, the results of high and low ${N}_{3D}^{{peak}}$ are listed in Table 1.

**Table 1:** 1D FEL parameters with *K*_0_ = 1.4, *λ*_*u*_ = 25 mm

Case a	*ϵ* (MeV)	*λ*_1_ (nm)	*ρ*^*a*^	${L}_{G0}^{a}$ (mm)	${L}_{u}^{a}$ (m)
a1	100	640	0.18	6	0.12
a2	500	26	0.062	18	0.37
a3	1000	6.5	0.039	29	0.59

Case b	*ϵ* (MeV)	*λ*_1_ (nm)	*ρ*^*b*^	${L}_{G0}^{b}$ (mm)	${L}_{u}^{b}$ (m)
b1	100	640	0.012	94	1.9
b2	500	26	0.0042	274	5.5
b3	1000	6.5	0.0026	435	8.7

^**a**^*N*_*3D*_^*peak*^ = 9.0 × 10^21^ m^-3^

^**b**^*N*_*3D*_^*peak*^ = 2.8 × 10^18^ m^-3^

The Pierce parameters drop by one order of magnitude in the lower ${N}_{3D}^{{peak}}$ cases. The expansion of the electron transverse size could be a result of space charge effect, energy spread, imperfect beamline design and implementation, etc. While installing the undulator as close as possible to the plasma source point might be beneficial, the lack of strong focusing magnet and the intense drive laser are the obstacles. Being able to achieve a strong focusing force, plasma optics^68,69^ could be a candidate to accomplish such an extremely compact LWFA FEL set-up. Although the emittance and electron energy spread were not discussed above, the knowledge of 3D density of the electron bunch is still beneficial for the estimation of the gain process. In fact, not only the value ${N}_{3D}^{{peak}}$, but also the 3D shape affects the lasing process. We plan to conduct such research in the future.

Based on our study, a single-shot *N*_3*D*_ detector can be implemented at any position of the beam transportation line. Such a detector could be helpful to the construction of a compact FEL source: (i) By monitoring the *N*_3*D*_ just before the undulator, the highest ${N}_{3D}^{{peak}}$ can be achieved via the tuning of the laser plasma parameters and the beam focusing optics; (ii) If a laser electron acceleration system can only work stably in a certain range of electron energies and *N*_3*D*_, one can choose a suitable undulator specifications $\left({K}_{0},{\lambda }_{u}\right)$ to achieve a higher gain when the space and resources are limited, at the beamline design stage before manufacturing and installation of the undulator.

## Discussion

In conclusion, an electro-optic 3D snapshot of the laser wakefield accelerated electrons was conducted at a position 7 cm after the plasma source, by simultaneously performing OTR imaging and EO spatial decoding. The detailed structures were retrieved using numerical calculations assisted by genetic algorithm. The electron bunch was observed to have a transverse size < 30 micrometers. The current profile had a multi-peak shape. A sub-10 fs structure with a peak above 1 kA was demonstrated. The electron bunch had a peak 3D number density of ${N}_{3D}^{{peak}}$ ~ 9 × 10^21^ m^-3^. Such a detection can be performed at any position in a beam transport line for future applications. The results and methodologies in this article could be useful in studies of accelerators, high-power lasers, and terahertz optics.

## Materials and methods

### The validity of claiming the single-shot measurement

**Fig. 5:**
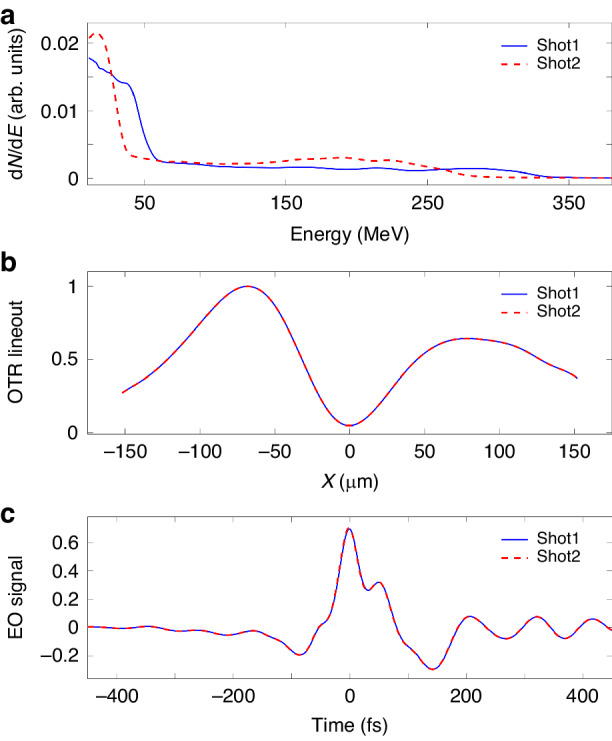
**COTR and EO signals from different electron energy distributions**. The blue and red curves denote the results of shot “1” and “2”, respectively. **a** The normalized experimental electron energy spectra of two shots. The overall electron numbers were normalized to 1. **b** The normalized horizontal COTR lineouts at “*Y* = 0”. **c** The EO signals of the two shots using the same current profile

By using a mixture gas (*H*_*2*_:*N*_*2*_) = (99:1) and a gas profile with a mild down ramp (see details in the “Supplementary information”), electron beams with pointing fluctuation less than 2 mrad (*rms*) and relatively stable energy spectra were generated. Here, we randomly choose 2 energy spectra to calculate the OTR profile and EO signal, using the 3D density profile from the best solution, as illustrated by Fig. 4. We found that, for the variation level of the electron energy spectra in our experiment, the OTR images and EO signals barely have differences, as shown in Fig. 5. This calculation supports the claim that 3D density reconstruction was a single-shot measurement in our experiment.

### The probe laser shape inside the EO crystal

**Fig. 6:**
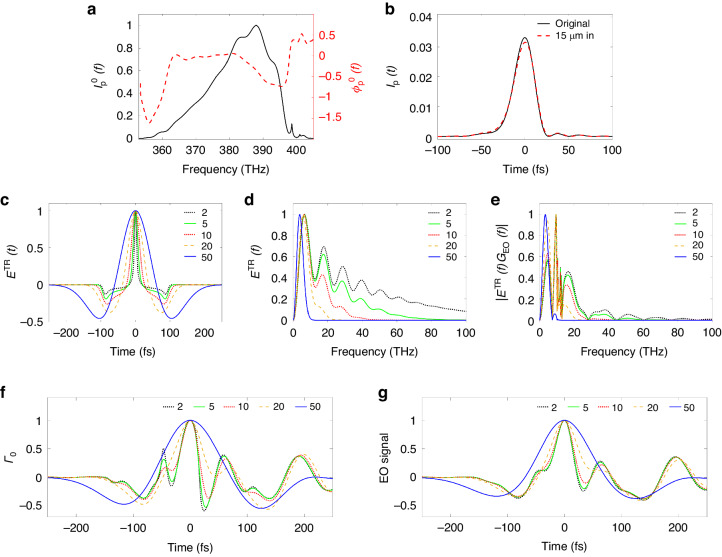
**Details of the EO calculation**. The information of probe laser is illustrated by **a,** **b**. **a** The original probe laser profile in frequency domain. The intensity *I*_*p*_^0^(*f*) (normalized with peak to 1) and phase *ϕ*^0^_*p*_(*f*) are denoted by black and red curves. **b** shows the shapes of the probe intensity profiles at propagation depths of 0 (black) and 15 (red) µm inside the EO crystal. The curves are normalized with constant energy. **c** The temporal profiles of the TR field incident onto the EO crystal. (black, green, red, yellow, blue) correspond to (2, 5, 10, 20, 50) fs, respectively. **d** The incident TR field in frequency domain. **e** The effective TR field in frequency domain considering EO geometric function *G*_*EO*_. **f** The phase retardations *Γ*_*0*_ with an infinitely short probe laser. **g** The EO signals including the temporal profile of the probe laser. Electrons with bunch durations of {2, 5, 10, 20, 50} fs were calculated. All the results in (**c**–**g**) are normalized with a maximum value of 1. The field information was picked at spatial point (0, -200) µm in the image plane

The final signal shape is related with the temporal profile of the probe laser. To calculate the EO signal shape, the initial probe laser spectrum ${I}_{p}^{0}\left(\omega \right)$ and phase ${\phi }_{p}^{0}\left(\omega \right)$ were detected using a self-referenced spectral interferometer (Wizzler, Fastlite Inc.). The duration of the probe laser was optimized to < 11 fs (*rms*) and measured by inserting a pick-up mirror between the polarizer “S” and the EO crystal in Fig. 1. The initial spectral intensity and phase of the probe laser were illustrated in Fig. 6a. The probe laser pulse shape *I*_*p*_ inside the crystal was determined by calculating the group-delay dispersion (GDD). The frequency phase of the probe laser at a propagation distance of *z* can be calculated by ${\phi }_{p}\left(\omega \right)=k\left(\omega \right)z$, where $k\left(\omega \right)={k}_{0}+{k}_{1}\varDelta \omega +{k}_{2}\varDelta {\omega }^{2}/2$. $\varDelta \omega =\left(\omega -{\omega }_{0}\right)$, where *ω*_0_ is the center angular frequency. ${k}_{0}={\omega }_{0}n\left({\omega }_{0}\right)/c$, ${k}_{1}={dk}/d\omega {{\rm{|}}}_{\omega ={\omega }_{0}}$ and ${k}_{1}={d}^{2}k/d{\omega }^{2}{{\rm{|}}}_{\omega ={\omega }_{0}}$ are the zero, first and second order dispersions around *ω*_0_. The temporal shape of the probe laser inside the EO crystal is then achieved by Fourier transformation. The probe profiles ${I}_{p}\left(t\right)$ at propagation depth of “0” and 15 μm are illustrated in Fig. 6b. For a very thin crystal, the change of the probe shape is quite small. The averaged probe shape achieved by separating the 30 μm GaP crystal to 120 slices was used to eliminate the numerical error. The accuracy of such an approach has been examined.

### Details of the EO calculation at femtosecond level

The EO signal calculation includes five steps: (i) The calculation of the incident TR field in frequency domain; (ii) The calculation of the effective TR field including the dispersive propagation of the TR pulse and Pockels effect; (iii) Calculation of the effective TR field in temporal domain; (iv) Calculation of the phase retardation with an infinitely “thin” probe laser; (v) Calculation of the EO signal considering the temporal broadening of the probe laser. Part of those processes have been elaborated in the main context of the article. Here, some details about the EO signal calculation are to be explained.

In the following calculation, the peak currents of the electron bunches are 1 kA. Although the signal intensities are different, we compare the shapes of the signals by normalizing the maximums to 1. Using the transverse electron profile measured in the experiment, the temporal profiles of the incident TR fields are calculated, as shown in Fig. 6c. The incident TR fields in the frequency domain are plotted as Fig. 6d. The shapes of the fields had obvious differences for electron bunch durations from 2 fs to 50 fs. The effective TR fields by including the geometric EO response function^50,51,65^ *G*_*EO*_ of a 30 μm GaP crystal are illustrated in Fig. 6e, where ${G}_{{EO}}=\frac{{r}_{41\left(\omega \right)}}{d}{\int }_{0}^{d}\exp \left[i\omega \left(\frac{N\left(\omega \right)}{c}-\frac{1}{{v}_{\parallel }}\right){dz}\right]$. The distributions in Fig. 6e show considerable differences in the high frequency range, which result in the observable differences of the phase retardation profiles *Γ*_0_, even when the electron bunches possess femtosecond bunch durations, as shown in Fig. 6f. Here, *Γ*_0_ is the phase retardation with a delta-function-like probe laser. However, since the EO signal is processed as ${I}_{{sig}}=\left[\cos \left(4{\theta }_{2}\right)-\cos \left(\varGamma +4{\theta }_{2}\right)\right]/\left[1-\cos \left(4{\theta }_{2}\right)+2{\delta }_{{ext}}\right]$ with $\varGamma ={\varGamma }_{0}\star \bar{{I}_{p}}$, the signal shapes differ from *Γ*_0_, as shown in Fig. 6g. Figure 6g shows a larger temporal area than Fig. 3d. When the electron bunch duration is larger than 50 fs, the oscillations barely appear in the EO signal, indicating that the electron temporal profile in the experiment must have structures less than a few tens of femtoseconds.

**Fig. 7:**
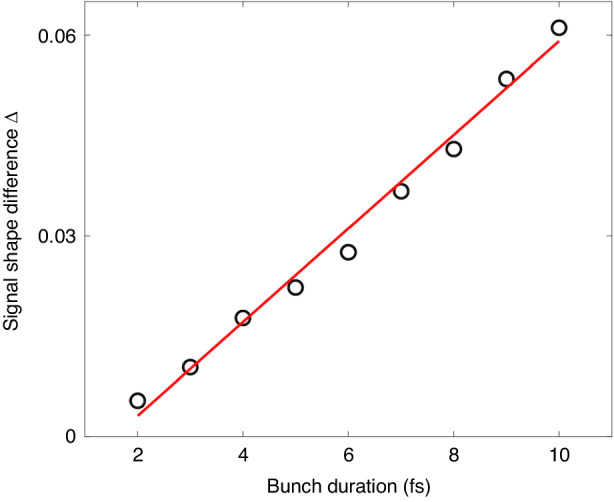
**Shape differences of normalized EO signals with femtosecond electron bunch durations**. The black circles denote the differences relevant to the EO signal shape from the electron bunch with a temporal duration of 1 fs. The red line illustrates a linear fitting of the scattered data

The approach in this work is not just a read-out of the fitted duration of the main peak of the raw EO signal, but rather, to calculate the shape of the EO signals. To quantitatively confirm whether the signals have differences when the electron bunches possess temporal structures < 10 fs, a signal difference function was defined as: $\varDelta =\sqrt{{\sum }_{{i}_{1}}{\left[{I}_{{sig}}\left({i}_{1}\right)-{I}_{{sig}}^{1{fs}}\left({i}_{1}\right)\right]}^{2}/{N}_{{sample}}}$, *i*_1_ denotes the indices of the signal array and *N*_*sample*_ is the size of the array. The dependence of *Δ* on bunch duration is plotted in Fig. 7. It can be readily seen that the shape difference grows almost linearly with the electron bunch duration.

### Details about the Genetic algorithm

The set-up of the GA for OTR are: the gene is arranged as ${geneOTR}=\{\varLambda ,{\sigma }_{x},{\sigma }_{y},\varphi ,{C}_{x},{C}_{y},{P}_{x},{P}_{y}\}$; the chromosome is ${chromoOTR}=\{{geneOTR},\ldots \times 6\}$. Each gene corresponds to a transverse distribution with a 2D super-gaussian shape described by

6 $${g}_{\perp }^{0}=\Lambda \exp \left[-{\left(\frac{d{x}_{1}^{2}}{2{\sigma }_{x}^{2}}\right)}^{{P}_{x}}-{\left(\frac{d{y}_{1}^{2}}{2{\sigma }_{y}^{2}}\right)}^{{P}_{y}}\right]$$

*Λ* is a positive number that describes the weight. (${\sigma }_{x},{\sigma }_{y}$) denote the sizes. *φ* is the rotation angle of the *x*-axis. (${C}_{x},{C}_{y}$) indicate the center positions. (${P}_{x},{P}_{y}$) denote the power numbers of the super-Gaussian. ${dx}=x-{C}_{x}$ and ${dy}=y-{C}_{y}$. A coordinate transformation of axis rotation was used: $d{x}_{1}=\cos \varphi \cdot {dx}+\sin \varphi \cdot {dy}$ and $d{y}_{1}=-\sin \varphi \cdot {dx}+\cos \varphi \cdot {dy}$. The variation ranges of each element of the *geneOTR* are {(0, 1), (2, 50) μm, (2, 50) μm, (-*π*/2, *π*/2), (-60, 60) μm, (-30, 30) μm, (0.8, 1.5), (0.8, 1.5)}, based on the bunch sizes calculated from the “1 Gauss” model. The overall transverse profile is ${g}_{\perp }=\sum {g}_{\perp }^{0}$. The fitness function is defined as: ${f}_{{OTR}}={\sum }_{{i}_{1}{i}_{2}}{\left[{I}_{{OTR}}^{\exp }\left({i}_{1},{i}_{2}\right)-{I}_{{COTR}}^{{GA}}\left({i}_{1},{i}_{2}\right)\right]}^{2}$, where ${I}_{{OTR}}^{\exp }$ and ${I}_{{COTR}}^{{GA}}$ are the normalized OTR profiles of the experiment and GA calculation, respectively. (*i*_1_, *i*_2_) are the indices of the 2D matrices.

In the experimental OTR image, the maximum intensity was 12.7 times stronger than the signal intensity at (*x*, *y*) = (0, 0). Such a result indicated that the coherent OTR feature contributed to a major part of the OTR image. For a quicker convergence of the GA calculation and to reproduce the main feature of the OTR, we set a cut-off threshold at 30% of the maximum for the results from both experiment and calculation. The feasibility of such an approach has been confirmed (see details in the “Supplementary information”).

The GA set-up for the EO calculation is: ${geneEO}=\{\varLambda ,{C}_{t},{\sigma }_{t},P\}$, ${chromoEO}=\{Q,{geneEO}\times 6\}$. The individual temporal distribution is

7 $${g}_{z}^{0}=\Lambda \exp \left\{-{\left[\frac{{\left(t-{C}_{t}\right)}^{2}}{2{\sigma }_{t}^{2}}\right]}^{P}\right\}$$

Each ${geneEO}$ has four elements: *Λ* (weight), *C*_*t*_ (temporal center), *σ*_*t*_ (duration), and *P* (power number). We set a variation range of {(0, 1), (-50, 50) fs, (*σ*_*l*_, 100) fs, and (0.8, 1.5)}. *σ*_*l*_ denotes the lower limit of the duration. The total charge *Q* varies between 10 and 200 pC. The overall longitudinal profile was calculated as ${g}_{z}=\sum {g}_{z}^{0}$ and then normalized. The fitness function is defined as: ${f}_{{EO}}={\sum }_{{i}_{1}}{\left[{I}_{{sig}}^{\exp }\left({i}_{1}\right)-{I}_{{sig}}^{{GA}}\left({i}_{1}\right)\right]}^{2}$, where *i*_1_ indicates the indices of the arrays, ${I}_{{sig}}^{\exp }$ and ${I}_{{sig}}^{{GA}}$ correspond to the experimental and GA calculation results. The fitness was calculated in a range of (-175, 175) fs in the EO signal to reproduce temporal structures around the main peak. With a plasma density of ~ 10^18 ^cm^−3^ (see details in the “Supplementary information”), the centers of the individual super-Gaussians were set to vary in a range of 100 fs to reproduce the temporal structure around the first bucket of the wake wave.

**Fig. 8:**
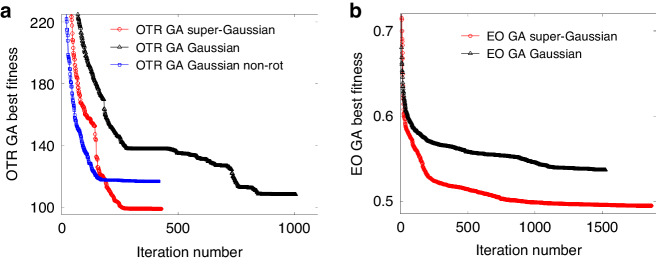
**Comparison of the iteration behaviors of different kinds of gene structures in the GA calculations. a** The results of OTR GA calculations. The red circles denote the best fitness values in each generation when using the super-Gaussian model as described by Eq. (6). The black triangles denote the results of the model using a Gaussian distribution and coordinate rotations. The blue squares correspond to the model using a simple Gaussian profile and no coordinate rotations. **b** The results of EO GA calculations. The red circles and black triangles denote the best fitness values in each generation when using the super-Gaussian and Gaussian models, respectively

The algorithms of GA have been extensively studied for decades^70^. Our numerical efforts concentrated on the design of the calculation code of the OTR and EO with high efficiency and the design of the structures of the genes and chromosomes. The GA calculation in this work was conducted by using the genetic algorithm applications provided by MathWorks. The roulette wheel selection was applied as selection function. To accelerate the calculation, the fitness function codes dealing with the OTR imaging and EO signal generation were vectorized in advance. The functions were evaluated in parallel during the iterations. The population was composed of 100 chromosomes. The calculation stopped when 50 stall generations happened. The stopping condition ensured the evolution of the fitness value reaching a steady state, as shown in Fig. 8. A smaller fitness value indicates a better solution. For both the OTR GA and EO GA calculation, we performed scanning on the gene number. Based on the fitness evolution trend, we chose gene numbers of {6, 6} for the {OTR, EO} GA calculations, respectively, as a compromise between better fitness and less calculation time.

The application of the super-Gaussian (SG) feature enhanced the diversities of the genes. For GA, the diversity of the gene structure is important. Since the selection tends to choose the solutions with better fitness values during iterations, the diversity of the genes determines the best solution in the final generation. We compared the GA iteration trends of different gene structures.

For OTR GA calculation, the simplest structure would be a pure Gaussian profile as ${g}^{{Gaussian},{non}-{rot}}=\varLambda \exp [{(x-{C}_{x})}^{2}/(2{\sigma }_{x}^{2})-{(y-{C}_{y})}^{2}/(2{\sigma }_{y}^{2})]$, the best fitness value in each generation is plotted as blue squares in Fig. 8a. Due to the lack of diversity and limited parameters, although the iteration converges quickly, the final fitness value is high (bad). By adding the rotating factor, the gene becomes ${g}^{{Gaussian}}=\varLambda \exp [-d{x}_{1}^{2}/(2{\sigma }_{x}^{2})-d{y}_{1}^{2}/(2{\sigma }_{y}^{2})]$, where $d{x}_{1}$ and $d{y}_{1}$ are the coordinates after rotation transformation. The final fitness value is smaller (better), as denoted by the black triangles. Finally, by modifying the Gaussian shape to a super-Gaussian as described by Eq. (6), the diversity of the bunch shape increases. The iteration trend is denoted by the red circles. The final fitness value was the best. For EO GA calculation, a comparison was also conducted between the “SG” model and the simple Gaussian model, as shown in Fig. 8b. The solution from “SG” model had a better fitness value in the end.

### Supplementary information

Supplemental Material
