# Supplementary material for: Electro-optic 3D snapshot of a laser wakefield accelerated kilo-ampere electron bunch
Source: Light Sci Appl. 2024 Apr 8;13:84. doi: 10.1038/s41377-024-01440-2 (PMC10999425; doi:10.1038/s41377-024-01440-2)
Supplement: Supplementary file 1 — Supplemental Material [file 41377_2024_1440_MOESM1_ESM.pdf]

Supplementary Information for

Electro-optic 3D snapshot of a laser wakefield accelerated kilo-ampere electron bunch

Kai Huang,[1,2*] Zhan Jin,[2,3] Nobuhiko Nakanii,[1,2] Tomonao Hosokai,[2,3] and Masaki Kando[1,2]

[1] Kansai Institute for Photon Science (KPSI), National Institutes for Quantum Science and Technology (QST), 8-1-7 Umemidai, Kizugawa-city, Kyoto, 619-0215, Japan

[2] Laser Accelerator R&D, Innovative Light Sources Division, RIKEN SPring-8 Center, 1-1-1 Kouto, Sayo, Hyogo, 679-5148, Japan

[3] SANKEN, Osaka University, 8-1 Mihogaoka, Ibaraki, Osaka, 567-0047, Japan

[*]Corresponding author: huang.kai@qst.go.jp

This is a supplementary file with the supportive information of plasma density, simulation, details of OTR and EO calculations, and other issues in the experiment.

1. The plasma density and possible time gap between two bunches

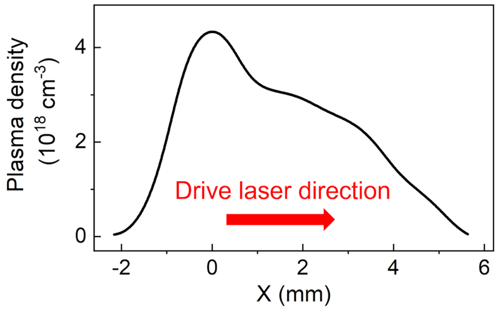

Figure S1 The electron plasma density profile. The red arrow denotes the propagation direction of the drive laser.

The gas profile from the supersonic nozzle was measured offline by using a Mach-Zehnder interferometer. The electron plasma density profile can be found in Fig. S1. The electron plasma density was calculated considering that the 2 electrons from the hydrogen molecule were ionized. In the experiment, we have used a slit nozzle. The gas profile was assumed to be uniform in the transverse direction. The density profile has a peak at 4×10^{18} cm^{-3} at the entrance, followed by a mild down ramp. Major part of the density profile is at the level of $\sim 10^{18}$ cm^{-3}, corresponding

to plasma wavelength of 33 μm (100 fs). Considering the bucket is elongated at the tail of the gas, current peaks with time gap $<$ 100 fs should reside in the same bucket of the plasma wave, as can be found in the GA reconstructed current profile.

2. Periodical density modulation of electrons often occurs in ionization injection

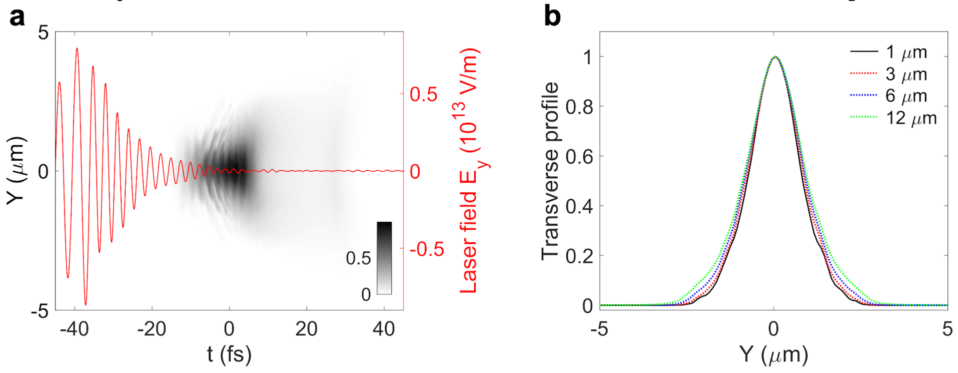

Figure S2 The PIC simulation results. **a** shows the lineout of transverse electric field of the drive laser (red), and the normalized 2D density distribution of electrons ionized from nitrogen K-shell (gray). **b** Integrated transverse beam profile along the longitudinal direction (time direction). The normalized transverse profile with longitudinal accumulation ranges of {1, 3, 6, 12} μm are plotted out, corresponding to temporal ranges of {3.3, 10, 20, 40} fs, respectively.

To qualitatively explain the validity of using the COTR profile to reconstruct the electron transverse profile in the case of ionization injection, a 2-dimensional (2D) particle-in-cell simulation by using the EPOCH code[1] has been conducted. The plasma density parameters were set similar as those in the experiment. The results were retrieved at a timing when the electrons leave the plasma. The electrons did not expand in the longitudinal and transverse direction at such a position. The background electrons were not injected. The electrons ionized from K-shell of nitrogen had modulated profiles, as shown in Fig. S2a. The sub-femtosecond modulation had periodic structures but did not localize at a certain spatial point. Figure S2b shows the normalized transverse beam profiles by longitudinal accumulation in ranges of {1, 3, 6, 12} μm around the peak of the density. Even with larger accumulation areas, the transverse profile kept similar shapes. No density singularities were observed. When the laser has a slowly varying transverse envelope, the transverse profiles of the electrons ionized from K-shell have similar shapes at different longitudinal locations.

The simulation indicated that, although the OTR would have a coherent pattern due to the sub-fs modulation, to some extent, the primary features of integrated electron beam transverse profile could be reconstructed using a COTR calculation.

3. Simplification of the OTR calculation and transverse resolution

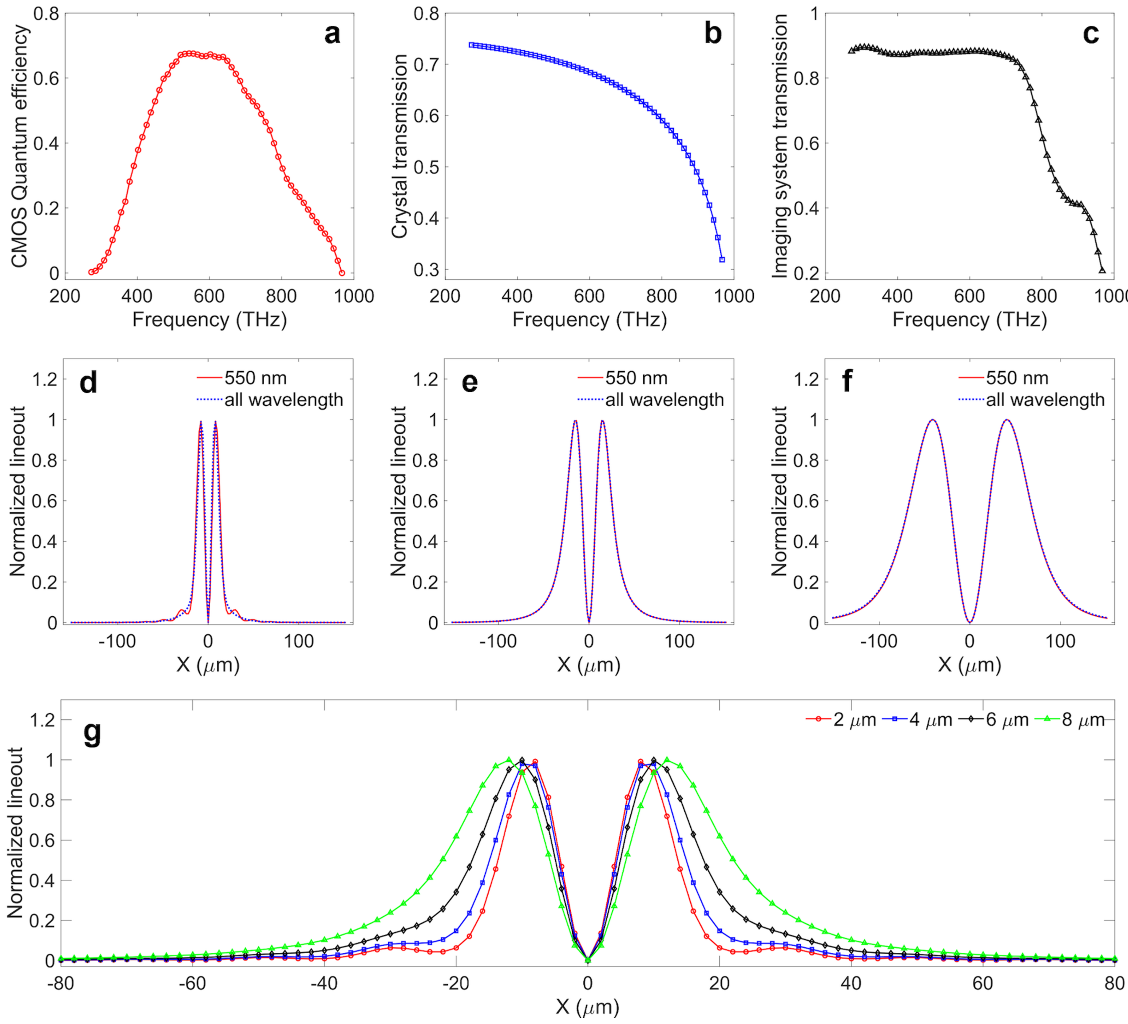

Figure S3 Discussion on the OTR imaging. **a** The interpolated frequency dependent quantum efficiency $QE(f)$ of the OTR camera. **b** The amplitude transmission rate of the GaP crystal. **c** The intensity transmission rate of the optics in the imaging system. The normalized lineouts of COTR profiles for transverse electron bunch sizes of {2, 10, 30} µm are illustrated in **d–f**. The blue dots illustrate the results from the model of "all wavelength". The red curves are the results from the single wavelength model of "550 nm". **g** The normalized lineouts of COTR profiles for transverse electron bunch sizes of {2, 4, 6, 8} µm.

In the experimental set-up, a bandpass filter was absent for the OTR imaging. However, we demonstrate that the OTR imaging calculation can be simplified using a single wavelength of $\overline{\lambda} =$ 550 nm. The camera used for OTR imaging was implemented with a Sony IMX264 CMOS chip (Pregius gen2). The quantum efficiency (QE) data could be found at "https://scientificimaging.com/knowledge-base/qe-curves-for-cmos-imagers/". We conducted the TR calculation in a uniformly distributed frequency domain. The interpolated QE(f) is plotted in Fig. S3a. The QE outside the plotted frequency region is almost zero. The field transmission rate

of the GaP crystal was calculated as $T^{crystal} = T_1 \times T_2$, where $T_1 = 2/(1 + n_{GaP})$ is the amplitude transmission coefficient on the front side of the crystal, $T_2 = 2n_{GaP}/(1 + n_{GaP})$ is the amplitude transmission coefficient on the rear side of the crystal. The refractive index in the visible range was[2] $n_{GaP} = \sqrt{\left(2.68 + 6.4\lambda[\mu m]^2/(\lambda[\mu m]^2 - 0.0903279)\right)}$. The amplitude transmission rate $T^{crystal}(f)$ is plotted in Fig. S3b, where $f = c/\lambda$. The transmission of the overall imaging system should include the transmissions of an imaging lens, a silver reflection mirror (not shown in the experiment set-up), and a BK7 window on the vacuum chamber wall. The overall transmission rate T^{optics} of the imaging system was plotted in Fig. S3c.

When including all the wavelength components λ_i in the responsive range of the camera and all the electron energy components with weight of χ_j, the relative COTR distribution should be calculated as follows:

The field point spread functions of a certain wavelength were calculated as: $FPSF^i_{x,y} = T_i^{crystal} \sum_j \chi_j \, E^0_{x,y}(\lambda_i, \gamma_j)$. The relative COTR intensity including all wavelengths was $I^{COTR}_{all} = \sum_i Q \, E_i T_i^{optics} \left(\left|g_\perp * FPSF^i_x\right|^2 + \left|g_\perp * FPSF^i_y\right|^2\right)$, and can be extended as:

$$I^{COTR}_{all} = \sum_i Q \, E_i T_i^{optics} \left(T_i^{crystal}\right)^2 \times \left[\left|g_\perp * \sum_j \chi_j \, E^0_x(\lambda_i, \gamma_j)\right|^2 + \left|g_\perp * \sum_j \chi_j \, E^0_y(\lambda_i, \gamma_j)\right|^2\right] \quad (1)$$

The calculation of Eq. (1) is time consuming. From Eq. (1), an averaged wavelength can be defined as: $\bar{\lambda} = \sum_i Q \, E_i T_i^{optics} \left(T_i^{crystal}\right)^2 \lambda_i / \sum_i Q \, E_i T_i^{optics} \left(T_i^{crystal}\right)^2$. The calculated averaged wavelength was $\bar{\lambda} \sim 550$ nm. The relative COTR intensity by only calculating one wavelength was:

$$I^{COTR}_{\bar{\lambda}} = \left|g_\perp * \sum_j \chi_j \, E^0_x(\bar{\lambda}, \gamma_j)\right|^2 + \left|g_\perp * \sum_j \chi_j \, E^0_y(\bar{\lambda}, \gamma_j)\right|^2 \quad (2)$$

The calculation of one wavelength takes much less time. To confirm the accuracy of this treatment, we did comparison calculations for electron transverse bunch sizes of {2, 10, 30} μm with the models of "550 nm" and "all wavelength". The normalized lineouts of the COTR profiles along the "X" axis at Y = 0 were plotted as Fig. S3(d–f). We observed that the two models had negligible differences for all electron bunch sizes. This comparison suggested that it was adequate to use a single wavelength of 550 nm to calculate the COTR profiles.

For the using a lower limit of transverse size of 2 μm in the GA calculation, the reasons are: (i) Calculations showed that there were distinguishable differences even for transverse electron bunch sizes at the level of a few micrometers, as shown in Fig. S3g; (ii) The spatial resolution of the 2D mesh in the calculation was 2 μm; (iii) The lower limit of 2 μm does not necessarily result in a spatial spike. It just enriched the diversity of the achievable transverse shapes.

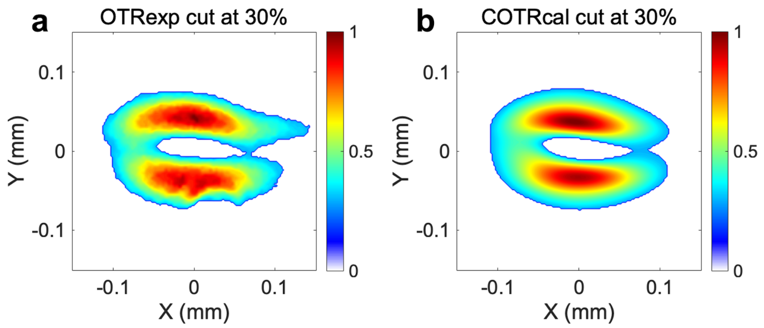

Figure S4 The normalized OTR images with a cut-off at 30 % of the maximum. a and **b** are the results from experiment and GA calculations, respectively. The results are normalized with maximum to 1.

4. The feasibility of COTR calculation for the reconstruction of the transverse profile

In the experimental OTR image, the maximum intensity was 12.7 times stronger than the signal intensity at (x, y) = (0, 0). Such a result indicated that the coherent OTR feature contributes to a major part of the OTR image. For a quicker convergence of the GA calculation and to reproduce the main feature of the OTR, we set a cut-off threshold at 30% of the maximum for the results from both experiment and calculation. The normalized OTR results after cut-off are plotted in Fig. S4. Since the incoherent component was magnitudes weaker than the coherent component, such a process should be reasonable. The main structure of the experimental OTR was well reproduced by using the coherent OTR calculation, as shown in Fig. S4.

Although very short modulations due to ionization-injection might exist, it cannot be recognized by the 30 μm GaP crystal in our experiment. In the manuscript, we have elaborated that "…The lateral analysis focuses on the envelope of the absolute current profile formed by the majority of the electrons."

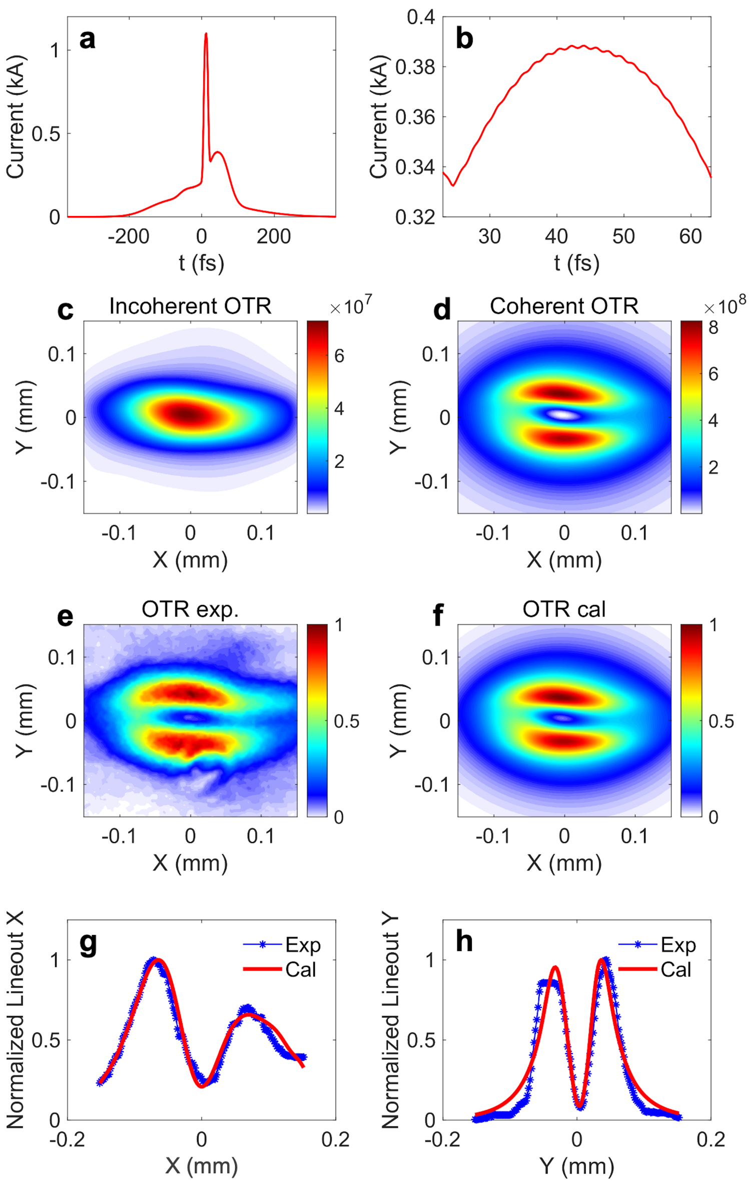

Figure S5 OTR signal calculations with small current modulations. a The current profile in a broad temporal range. **b** The zoomed current profile showing very small modulations. **c** and **d** show the relative OTR intensity profiles with arbitrary units. **e** is the normalized experimental OTR image (max to 1). **f** is the normalized OTR image from the modulated current profile including both the coherent and incoherent components. **g** The normalized lineout along the horizontal line of "y = 0" in **e** and **f**. **h** The normalized lineout along the vertical line of "x = 0" in **e** and **f**.

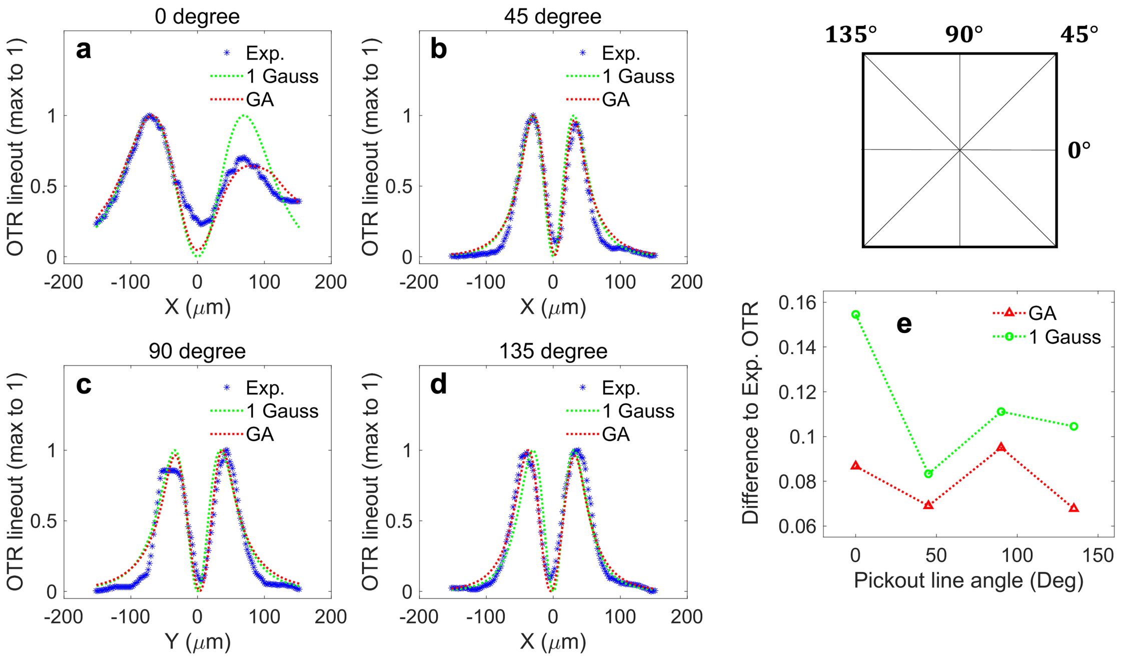

Figure S6 (a–d) The lineouts comparisons along the lines crossing the center of OTR image with difference angles. **e** shows the standard deviation of the lineout differences between the calculated signals and the experimental OTR.

To explain that such small modulations might be the reason of the formation of the COTR pattern in the experiment. We conducted a calculation by modifying the current profile achieved from the GA-EO calculations. Very mild oscillations were added to the envelope, which cannot be distinguished in a macroscopic view, as shown in Fig. S5a. In a zoomed temporal range, one can observed the oscillations, as shown in Fig. S5b. The incoherent and coherent image formed by such a current profile are plotted as Fig. S5c and d, respectively. The color scales are in arbitrary unit. The maximum value of the COTR is more than 10 times stronger than that from the incoherent OTR. As a comparison, the normalized experimental OTR and calculated OTR are plotted in Fig. S5e and f, respectively, where the calculated OTR include both the incoherent and coherent components. The normalized lineouts (max to 1) along the horizontal line "y = 0" and the vertical line "x = 0" in Fig. S5(e-f) are illustrated in Fig. S5(g–h). By including the incoherent component created by such a modulated current profile, the ratios of I_{max}/I_{min} of the calculated OTR almost coincide with the experimental signal. Although similar modulations were observed in a previous study[3], we do not intend to claim that the current profile in our experiment was exactly the same as in Fig. S5(a–b).

The lineouts along the lines crossing the center of the OTR image with difference angles are plotted in Fig. S6(a–d). In addition, the standard deviation of the lineouts were calculated as: $STD_{lineout} = \sqrt{\sum_{j_1}\left[I_{cal}(j_1) - I_{exp}(j_1)\right]^2/N}$, where j_1 is the index of the lineout array and N is the total number of elements in the array. In the lineouts along any angles, the solution from the "GA" shows smaller difference than that from "1 Gauss", indicating that GA greatly improved the similarity, as illustrated in Fig. S6e.

5. The single electron beam profile was confirmed by a transverse beam profile monitor 1 m downstream the source

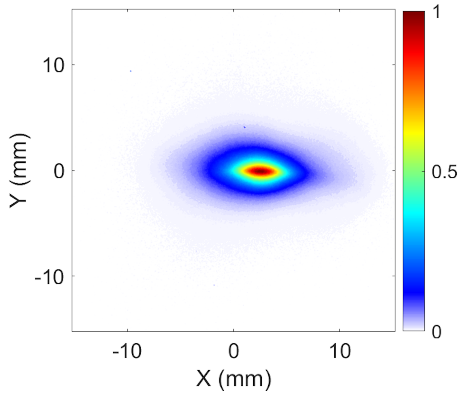

Figure S7 The typical electron transverse profile after 1 m propagation in a separate laser shot.

In addition to the TR-EO measurement, beam transverse profile was measured at 1 m downstream the plasma source when removing the OAP1 and metal foil, as shown in Fig. S7. This information was used as a cross-check of the electron profile. The beam shape was measured by using a DRZ: $Gd_2O_2S{:}Tb$ phosphor screen (Mitsubishi Chemical, DRZ-High). The electron bunch expanded due to space charge effect and inherent divergence. Still, the single beam (Gaussian-like) transverse profile indicated that the "donut" shape in the experimental OTR image resulted from the coherent imaging component, instead of two separate beams.

6. The impact of the absence of a central hole in OAP1

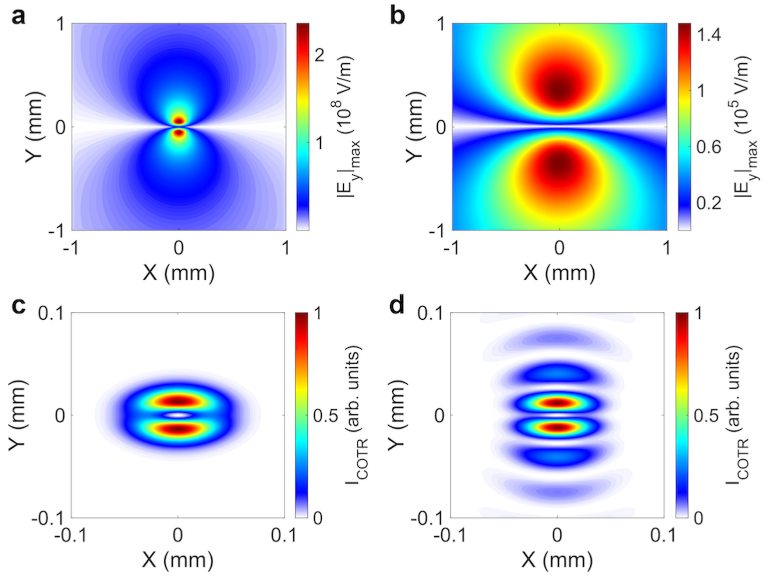

Figure S8 Discussion on the absence of a central hole in OAP1. **a** and **b** explain that the TR noise from OAP1 can be neglected. **a** $|E_y(t)|_{max}(X,Y)$ of TR emitted from the stainless-steel foil. **b** $|E_y(t)|_{max}(X,Y)$ of the TR created when the electrons hitting the OAP1. **c** and **d** show a comparison of the normalized COTR image by using OAP1 without a hole **c** and with a hole **d** (diameter of the hole is 3 mm). The magnification of the imaging in **a-d** is 1.

Due to the limitation of the experiment set-up, a bending magnet was not inserted between the metal foil and OAP1. Since OAP1 did not possess a central hole, TR could be created when the scattered electrons hitting the gold-coated surface of OAP1. Such an issue should be seriously treated when the electrons have high energy (GeV). However, when using a stainless-steel foil with thickness of 100 μm, the scattering of the electrons with low energies was severe[4] that majority of the electrons had beam size of several millimeters at the position of OAP1. The density of the electron bunch dropped by over a hundred time with an original beam size of merely a few tens of micrometers. We conducted a detailed calculation of the TR field created from OAP1, as shown in Fig. S8b. $|E_y(t)|_{max}(X,Y)$ is the 2D distribution of the peak value of the TR field in time domain. The calculations utilized the electron energy distribution in experiment, a transverse size of (28, 11) μm and an absolute current profile from the best solution of GA. The TR field contributed from OAP1 was 3 orders of magnitude smaller than the signal TR field from the stainless-steel foil. Thus, this issue can be ignored in our experiment. The details of the calculation method can be found in reference 4 of this supplemental file.

On the other hand, we did not use an OAP1 with a hole to avoid unwanted diffraction pattern in the COTR image. In the case without a hole, the diffraction factor is described as $f(\theta_m, \gamma, \zeta) \approx \gamma^{-1}K_1(\gamma^{-1}\zeta) - \zeta^{-1}J_0(\zeta\theta_m)$, where θ_m is the acceptance angle defined by the radius of OAP1. With a hole in the center, the diffraction factor is modified to $f(\theta_m, \theta_1, \zeta) \approx \zeta^{-1}[J_0(\zeta\theta_1) - J_0(\zeta\theta_m)]$, where θ_1 is the transmission angle defined by the radius of the hole. A comparison of the COTR images between "without hole" and "with hole" is shown in Fig. S8c and d. Both figures result from an electron bunch with energy of 50 MeV and transverse sizes of (28, 11) μm. In the case without a hole, as shown in Fig. S8c, the COTR image has a normal "donut" pattern. However, if the OAP1 has a hole with a diameter of 3 mm, multiple diffraction fringes appear, as shown in Fig. S8d. In such a case, the COTR pattern become quite messy. Such fringes make the reconstruction of the electron profile very difficult if the electron bunch has irregular structures.

References

[1]Arber, T. et al., Contemporary particle-in-cell approach to laser-plasma modelling, Plasma Physics and Controlled Fusion 57, 113001 (2015).

[2]Steffen, B. R. Electro-Optic Methods for Longitudinal Bunch Diagnostics at Flash. Ph.D. thesis, Hamburg. U, 2007, "https://bib-pubdb1.desy.de/record/84041/files/desy-thesis-07-020.pdf".

[3]Zarini, O. et al. Multioctave high-dynamic range optical spectrometer for single-pulse, longitudinal characterization of ultrashort electron bunches. Physical Review Accelerators and Beams 25, 012801 (2022).

[4]Huang, K. et al. Numerical study on femtosecond electro-optical spatial decoding of transition radiation from laser wakefield accelerated electron bunches. Physical Review Accelerators and Beams 26, 112801 (2023).
